# Complete Genome Sequence of *Bacillus* Phage Belinda from Grand Cayman Island

**DOI:** 10.1128/genomeA.00571-16

**Published:** 2016-10-13

**Authors:** Eileen F. Breslin, Jessica Cornell, Zachary Schuhmacher, Madison Himelright, Aya Andos, Ariel Childs, Ana Clem, Monica Gerber, Arissa Gordillo, Laith Harb, Reafa Hossain, Taylor Hutchinson, Isaac Miller, Edmund Morton, Ryan Walters, Destin Webb, Louise Temple

**Affiliations:** James Madison University, Harrisonburg, Virginia, USA

## Abstract

Soil from George Town, Grand Cayman Island, yielded the bacteriophage Belinda, isolated on *Bacillus thuringiensis* DSM 350. We present here the analysis of the complete genome sequence of 162,308 bp, with 298 predicted genes. The genome also contains three tRNA genes. Belinda belongs to the C1 cluster of *Bacillus* phages.

## GENOME ANNOUNCEMENT

Bacteriophage Belinda is a member of the *Myoviridae* family, isolated on *Bacillus thuringiensis* (*Bt*) DSM 350. *Bt* is a common soil microbe that acts as a natural predator for soil nematodes (1) but does not cause human disease. Belinda was discovered in 2015 in soil from George Town, Grand Cayman Island (global positioning system [GPS] coordinates 19°17′35.3″N 81°23′04.8″W), having been isolated after soil enrichment with *Bt* DSM 350. Belinda and similar phages isolated on *Bt* DSM 350 also infected *Bacillus thuringiensis kurstaki* at about the same rate but do not infect more distantly related *Bacillus* species, as reported for C1 phages isolated on *B. thuringiensis kurstaki* (2).

Plaque purification was followed by DNA preparation using standard methodology. Phage genomic DNA was sequenced by the Pittsburgh Bacteriophage Institute using Illumina sequencing. The assembly program Newbler (3) was used to produce a single contig with 50,000 reads and ~100-fold depth of coverage. Gene prediction was completed using GeneMark (4) and Glimmer (5). Belinda was autoannotated and refined using DNA Master (http://cobamide2.bio.pitt.edu/computer.htm). After analysis using protein BLAST (6), HHPred, and Conserved Domains Database, functions of proteins were predicted. tRNA genes were annotated and confirmed using ARAGORN (http://130.235.46.10/ARAGORN/).

Using BLASTn analysis (7), the closest relative found was *B. thuringiensis kurstaki* phage Zuko (accession no. KU737348), showing 90% identity and 95% coverage. Belinda was also similar to Hakuna (accession no. KJ489399) and other C1 cluster phages (2). Highly similar phages being isolated from widely separated geographical locations is interesting but not unprecedented.

The Belinda genome consisted of 162,308 bases, with a G+C content of 38.8%, and had a direct terminal repeat of 2,974 bp (8) based on the occurrence of the double-coverage region in the assembled contig (3). The terminal repeats include approximately 1,500 bases of noncoding sequence and two predicted hypothetical proteins. Thirty-nine genes encoding functional proteins were putatively identified. Of these, 11 structural proteins were identified, including tail fiber proteins, baseplate proteins, tail lysins, a prohead protease, and a major capsid protein. Lysis genes included three hydrolases and a holin. DNA binding proteins with potential to affect gene expression were identified (helix-turn-helix proteins and sigma factors). Eight DNA replication proteins were identified, including helicase, primase, and polymerase. The DNA polymerase is present in two segments, resulting from a frameshift, similar to several reported *Bacillus* phages where the polymerase gene is interrupted by introns or frameshifts (2). Ten proteins utilized in nucleic acid metabolism were identified, such as ribonucleotide-diphosphate reductase, adenylate kinase, thymidylate synthase, and nicotinamide phosphoribosyltransferase. Recombinase A and Holliday junction resolvase were also predicted. We isolated putative lysogens by picking within plaques and culturing; however, when tested, these bacteria were not resistant to reinfection, indicating that they were the original host strain. The chromosome also lacked any putative repressor genes, the presence of which would be expected in a temperate bacteriophage. Because other C1 phages are reported to be virulent, it is likely that Belinda is virulent.

### Accession number(s).

The complete genome of bacteriophage Belinda is deposited in GenBank under the accession no. KX147229.
